# Optimizing CNN for pavement distress detection via edge-enhanced multi-scale feature fusion

**DOI:** 10.1371/journal.pone.0319299

**Published:** 2025-04-09

**Authors:** Jinwen Wang, Xiaowei Li, Yong Xu, Zhiyu Zhou, Wenlin Wu, Zheng Li

**Affiliations:** 1 Department of Rail Transportation, Shandong Jiaotong University, Jinan, China; 2 Department of Shandong Railway Transit, Survey and Design Institute Co., Ltd., Jinan, China; Shandong University of Technology, CHINA

## Abstract

Traditional crack detection methods initially relied on manual observation, followed by instrument-assisted techniques. Today, road surface inspection leverages deep learning to achieve automated crack detection. However, in the domain of deep learning-based road surface damage classification, the heterogeneous and complex nature of road environments introduces significant background noise and unstructured features. These factors often undermine the robustness and generalization capability of models, thereby adversely affecting classification accuracy. To address this challenge, this research incorporates edge priors by integrating traditional edge detection techniques with deep convolutional neural networks (DCNNs). This paper proposes an innovative mechanism called Edge-Enhanced Multi-Scale Feature Fusion (EE-MSFF), which enhances edge information through multi-scale feature extraction, thereby mitigating the impact of complex backgrounds and improving the model’s focus on crack regions. Specifically, the proposed mechanism leverages classical edge detection operators such as Sobel, Prewitt, and Laplacian to perform multi-scale edge information extraction during the feature extraction phase of the model. This process captures both local edge features and global structural information in crack regions, thereby enhancing the model’s resistance to interference from complex backgrounds. By employing multi-scale receptive fields, the EE-MSFF mechanism facilitates hierarchical fusion of feature maps, guiding the model to learn edge information that is correlated with crack regions. This effectively strengthens the model’s ability to perceive damaged pavement features in complex environments, improving classification accuracy and stability. In this study, the model underwent systematic training and validation on both the complex-background dataset RDD2020 and the simple-background dataset Concrete_Data_Week3. Experimental results demonstrate that the proposed model achieved a classification accuracy of 88.68% on the RDD2020 dataset and 99.5% on the Concrete_Data_Week3 dataset, where background interference is minimal. Furthermore, ablation studies were conducted to analyze the independent contributions of each module, highlighting the performance improvements associated with the integration of multi-scale edge features.

## Introduction

With the rapid growth of transportation demands and accelerated urbanization, maintaining and enhancing road infrastructure has become a critical factor in ensuring economic development and improving the quality of life [1]. As the primary load-bearing layer of the transportation network, the condition of road surfaces directly impacts driving safety and traffic efficiency. However, due to prolonged exposure to vehicular loads, environmental factors, and seasonal changes, road surface deterioration has become increasingly prominent. Among these issues, road surface cracks—one of the most common forms of damage—pose a dual threat: they degrade road performance and accelerate the development of other forms of damage, such as water infiltration leading to foundation damage, ultimately shortening the lifespan of roads and increasing maintenance costs [2].

Traditionally, the detection and evaluation of road damage have relied heavily on manual inspections. This method is not only time-intensive and labor-intensive but also constrained by inspectors’ experience, subjective judgment, and working conditions, often failing to meet the standards of efficiency and accuracy [3]. Additionally, the periodic nature of manual inspections makes it difficult to monitor real-time changes in road conditions, leading to delayed responses to sudden or rapidly evolving damage and increasing repair costs [4]. Therefore, there is an urgent need for a more efficient, economical, and accurate method for detecting and evaluating road damage.

In recent years, computer vision has demonstrated significant advantages in image classification, gradually replacing labor-intensive manual inspections with more efficient and reliable solutions [5–7]. For instance, Gabor filters have been applied for crack detection, capable of identifying cracks of various orientations and types by processing images through a set of direction-specific filters. The filtered responses are subsequently thresholded and combined to delineate crack regions [8]. Additionally, the Canny edge detection operator has been employed to detect cracks on airport runways. This approach integrates Canny edge detection with morphological operations to automate measurements of crack length, width, and area, demonstrating the effectiveness of edge detection in crack analysis [9]. Similarly, edge detection algorithms combined with morphological operations have contributed to enhancing crack detection accuracy [10].

Despite their utility, traditional methods often face limitations in detecting cracks against complex road backgrounds, as they typically rely on hand-crafted gradient features or geometric assumptions. Deep convolutional neural networks (CNNs) effectively address these challenges [11]. By integrating edge detection with CNNs, further performance improvements can be achieved. For example, combining CNNs with Laplacian of Gaussian (LoG) edge detection enhances the detection accuracy of negative (non-crack) samples [12]. Incorporating edge detection strategies into deep learning architectures has also led to notable improvements in both accuracy and efficiency [13]. Moreover, detection accuracy has been further refined by replacing the Gaussian filter in Canny edge detection with a SUSAN (Smallest Univalue Segment Assimilating Nucleus) filter, resulting in reduced noise and improved identification of image edges and corners [14].

This study focuses on developing a novel road damage detection method with a particular emphasis on accurately identifying road cracks. The core innovation lies in introducing an Edge-Enhanced Multi-Scale Feature Fusion (EE-MSFF) mechanism within a deep CNN framework. By optimizing multi-scale feature extraction, the model enhances its ability to learn crack edge features, effectively suppressing interference from non-crack regions in complex road environments. Building upon a comprehensive analysis of crack detection challenges, the study specifically optimizes the ResNet deep CNN architecture to address the intricate task of crack classification, achieving notable accuracy improvements. While standard CNNs have been extensively used for image recognition, they often fall short in detecting small and irregular cracks in complex road environments due to insufficient attention to local details. To address this, a method that combines edge detection mechanisms with multi-scale feature fusion strategies is proposed. This design enables the model to capture a global understanding of the image while actively focusing on crack edges. By integrating edge-guided multi-scale feature modules into the deep network, the model is better directed to distinguish crack regions, reducing the impact of complex backgrounds and improving classification performance.

The contributions of this study are summarized as follows:

1. A novel EE-MSFF mechanism for multi-scale feature fusion is proposed, with emphasis placed on edge information in images to guide CNNs to focus on road crack regions.
2. Experiments on the RDD2020 dataset with complex backgrounds demonstrate that the model achieves an accuracy of 88.68% using this mechanism.
3. Ablation experiments, including comparative visualizations, validate the improvements in model performance, confirming the effectiveness of the proposed enhancements.
4. The proposed model is compared with various classical image classification methods on the RDD2020 dataset, showcasing its advantages and competitiveness in crack detection tasks.
5. Small-sample experiments on the Concrete_Data_Week3 dataset further highlight the model’s excellent small-sample learning capability and generalization performance.

## Materials and methods

### Experimental data preparation

In this experiment, two different datasets were used for validation. The first dataset is the road crack classification dataset RDD2020 [15] with a complex background. The second dataset is the road crack classification dataset Concrete_Data_Week3 [16] with a simple background.

**Fig 1:**
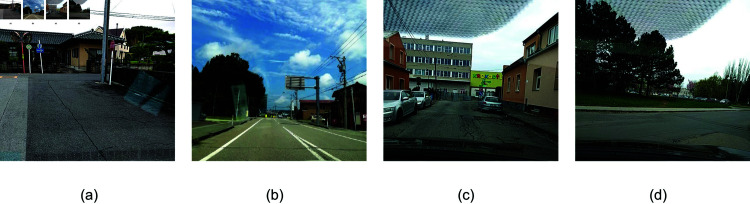
Images of different disease categories. (a) The longitudinal cracks. (b) The transverse cracks. (c) The alligator cracks. (d) The potholes. Republished from Reckless_Raccoon, RDD_2020 Dataset, which is available under a CC0 1.0 Universal Public Domain Dedication. The dataset can be accessed at: https://www.kaggle.com/datasets/recklessraccoon/rdd2020-yolov8-annotated-image-after/data.

The RDD2020 dataset is a publicly available road crack dataset. The dataset captures four types of road damage: Longitudinal Cracks (D00), as shown in Fig 1(a), Transverse Cracks (D10), as depicted in Fig 1(b), Alligator Cracks (D20), as illustrated in Fig 1(c), and Potholes (D40), as shown in Fig 1(d). However,it was found that after filtering, the sample images of Transverse Cracks (D10) were too few. To ensure the sufficiency of the training data, image augmentation techniques were employed to increase the number of images for this category. To ensure data diversity and improve the model’s generalization ability, a total of 4,174 road images from different regions were selected, including India, Japan, and the Czech Republic, as shown in Table 1. The dataset was split into a training set and a test set in a ratio of 0.8:0.2. For low-cost monitoring of road conditions by municipal authorities and road agencies, this dataset was captured using a vehicle-mounted phone, and it contains many images with non-road backgrounds, making the performance of this raw dataset on various models not particularly outstanding [17].

**Table 1:** RDD2020 dataset.

Class Label	Disease Type	Number of Samples
0	Longitudinal Crack	1040
1	Transverse Cracks	826
2	Alligator Cracks	1680
3	Potholes	628

The Concrete_Data_Week3 dataset is also available for open access. This dataset is a binary classification dataset with 40,000 images, including 20,000 images of road cracks and 20,000 images of intact roads. 1,000 images were selected as the training set and 200 images were selected as the validation set. Both the training and validation sets contain an equal number of Crack with and Non-Crack, as shown in Table 2.

**Table 2:** Concrete_Data_Week3 dataset.

Type	Crack	Non-Crack
Train	500	100
Valid	500	100

### Model architecture

**Fig 2:**
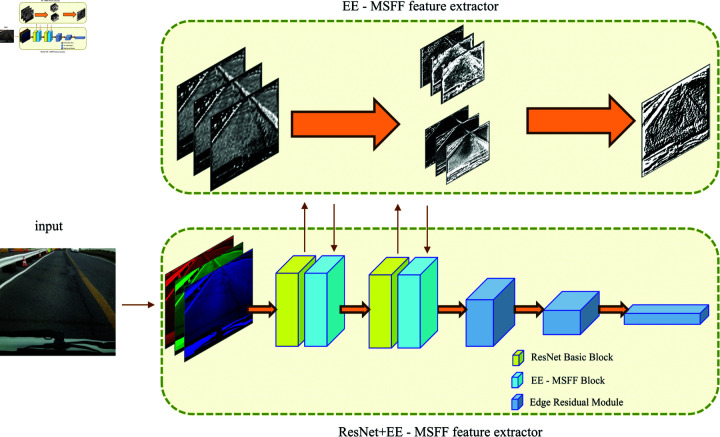
Overview of the proposed method.

As shown in Fig 2, the method framework used in this study mainly includes the following components: input data, edge information and multi-scale feature extraction, and image feature extraction using ResNet and the EE-MSFF mechanism. In the edge information and multi-scale feature extraction step, considering the importance of edge feature information, the edge feature statistics of the images are incorporated as input into the model. Specifically, three edge detection algorithms—Sobel operator, Prewitt operator, and Laplacian operator—are used to calculate the edge feature statistics for each image, and multi-scale features of the feature maps under different receptive fields are integrated. Finally, during the feature extraction process, the edge feature information is input into the ResNet residual module, forming an edge residual module with multi-scale edge feature information. In each EE-MSFF block, edge feature distribution information is introduced to enhance the model’s focus to edge features.

In the subsequent classification process, the output of the previous module is first down-sampled using average pooling. The reduced feature map is then flattened into a one-dimensional tensor and passed into a fully connected layer for classification prediction, resulting in an output tensor. Finally, an output layer with a number of neurons equal to the number of damage categories is introduced. By applying the softmax function to the output of the fully connected layer, the probability scores for each category can be calculated, thus determining the disease type to which the image belongs. In this study, ResNet18 was used as the residual network for edge information integration in the experiment.

### Integration of edge information

Deep convolutional neural networks have limited feature extraction capability for road damage areas, especially when affected by complex backgrounds. Therefore, the use of edge information as multi-scale features is proposed to assist the model in better recognizing road crack damage areas, which has been proven to be effective for recognizing target images with specific characteristics [18]. To integrate edge information, three different edge detection algorithms, namely the Sobel operator, Prewitt operator, and Laplacian operator, were combined. Below is an introduction to the edge algorithms introduced.

The Sobel operator is widely used in image processing [19]. It detects edges by calculating the gradient of image pixels in both the x and y directions, and its advantage lies in its ability to suppress noise. The gradient magnitude at each pixel is given by:


$$\begin{array}{ccc} G_{Sobel}=\sqrt{G_{Sobel,x}^{2}+G_{Sobel,y}^{2}} & & \end{array}$$ (1)

Where $G_{Sobel,x}$ and $G_{Sobel,y}$ represent the gradients in the *x* and *y* directions, respectively. The Sobel operator’s convolution template is as follows:


$$\begin{array}{ccc} G_{Sobel,x} & =\left[\begin{array}{ccc} -1 & 0 & 1\\ -2 & 0 & 2\\ -1 & 0 & 1 \end{array}\right] & \end{array}$$ (2)

$$\begin{array}{cc} G_{Sobel,y} & =\left[\begin{array}{ccc} -1 & -2 & -1\\ 0 & 0 & 0\\ 1 & 2 & 1 \end{array}\right] \end{array}$$ (3)

The Prewitt operator [20], similar to the Sobel operator, also detects edges by calculating the gradient of image pixels in the *x* and *y* directions. This method can accurately detect edge information in images, particularly for images with cracks. The gradient magnitude is given by:


$$\begin{array}{ccc} G_{Prewitt}=\sqrt{G_{Prewitt,x}^{2}+G_{Prewitt,y}^{2}} & & \end{array}$$ (4)

Where $G_{Prewitt,x}$ and $G_{Prewitt,y}$ represent the gradients in the *x* and *y* directions. The Prewitt operator’s convolution template is as follows:


$$\begin{array}{ccc} G_{Prewitt,x} & =\left[\begin{array}{ccc} -1 & -1 & -1\\ 0 & 0 & 0\\ 1 & 1 & 1 \end{array}\right] & \end{array}$$ (5)

$$\begin{array}{cccc} G_{Prewitt,y} & =\left[\begin{array}{ccc} -1 & 0 & 1\\ -1 & 0 & 1\\ -1 & 0 & 1 \end{array}\right] & & \text{(6)} \end{array}$$ (6)

The Laplacian operator [21] is a second-order derivative-based edge detection method. It detects edges by calculating the second-order derivatives of image pixels, which are sensitive to grayscale changes. This method quickly and accurately detects edges and excels in identifying details and texture information in images. The Laplacian at position (*x,y*) is given by:


$$\begin{array}{ccc} \nabla^{2}I=\frac{\partial^{2}I}{\partial x^{2}}+\frac{\partial^{2}I}{\partial y^{2}} & & \end{array}$$ (7)

Where *I* represents the intensity value of the image at pixel (*x,y*), and $\frac{\partial^{2}I}{\partial x^{2}}$ and $\frac{\partial^{2}I}{\partial y^{2}}$ are the second-order derivatives in the horizontal and vertical directions, respectively. Its convolution template operator as follows:


$$\begin{array}{ccc} G_{Laplacian}=\left[\begin{array}{ccc} 0 & 1 & 0\\ 1 & -4 & 1\\ 0 & 1 & 0 \end{array}\right] & & \end{array}$$ (8)

For a road damage image (size 3×224×224, pixel value range 0-255), the Sobel operator, Prewitt operator, and Laplacian operator are used to obtain learnable edge feature information from the image after the first convolution layer of the neural network.

The above three algorithms are applied to perform matrix operations, extracting multi-scale edge features from the image and combining them with feature maps of different receptive fields to obtain the EE-MSFF block. The EE-MSFF block is then introduced during the feature extraction process at the same depth to guide the model in assigning weights. As shown in Fig 2, in the ResNet+EE-MSFF feature extractor, the image feature extraction module is composed of a series of EE-MSFF BasicBlock modules forming a residual network structure. By combining these different structured blocks, their individual advantages are utilized to enhance the model’s feature extraction capabilities.

### EE-MSFF mechanism

#### Multi-Scale feature fusion.

Multi-scale feature fusion refers to the features obtained at different scales (such as different resolutions, different receptive fields, etc.) [22]. Different edge information is extracted from the image using different edge algorithms, such as the Sobel operator and the Prewitt operator, which are primarily based on the first-order derivative to calculate edges, and the Laplacian operator, which is based on second-order derivative edge detection methods. These algorithms can extract edge feature information at different scales. Convolution kernels of different sizes are also used to change the receptive field of the neurons, thereby obtaining features at different scales. Finally, the extracted features at different scales are fused to obtain the desired feature extraction module, i.e., the EE-MSFF Module.

#### EE-MSFF module.

**Fig 3:**
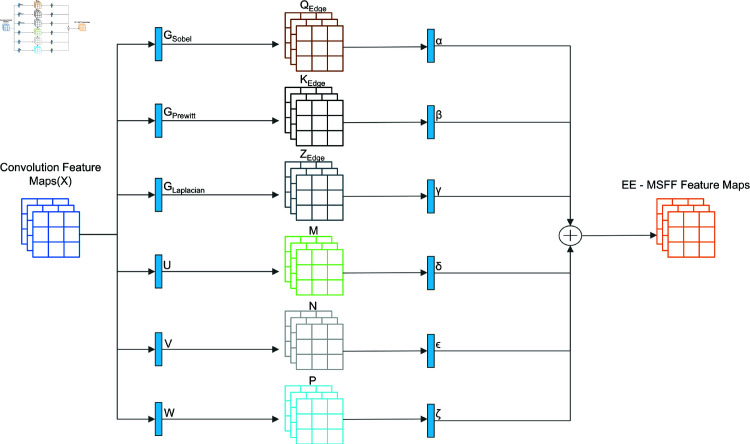
EE-MSFF module.

As shown in Fig 3, consider a feature map *X* with dimensions as follows:


$$\begin{array}{ccc} X\in {\mathbb{R}}^{b\;\times \;c\;\times \;h\;\times \;w} & & \end{array}$$ (9)

Where *b* represents the batch size, *c* the number of channels, and *h* and *w* the height and width of the feature map, respectively.

In the initialization phase of the EE-MSFF module, three independent convolution kernels, Eq (1), Eq (4), and Eq (8), are defined and expanded to C channels. These convolution kernels are used during forward propagation to extract edge features from the input feature map, resulting in new feature matrices, Eq (10), Eq (11) and Eq (12), as shown in the following equations:


$$\begin{array}{ccc} Q_{Edge} & =X*G_{Sobel} & \end{array}$$ (10)

$$\begin{array}{ccc} K_{Edge} & =X*G_{Prewitt} & \end{array}$$ (11)

$$\begin{array}{ccc} Z_{Edge} & =X*G_{Laplacian} & \end{array}$$ (12)

In Eq (10), Eq (11) and Eq (12), ∗ represents the convolution operation.

Next, features are extracted from the input feature map at different scales. Smaller convolution kernels capture local detail information, while larger kernels capture broader contextual information. This multi-scale approach enriches the model’s feature representation of the input data, improving its performance. The calculation process for the mapped M, N, and P is as follows:


$$\begin{array}{ccc} M & =X*U & \end{array}$$ (13)

$$\begin{array}{ccc} N & =X*V & \end{array}$$ (14)

$$\begin{array}{ccc} P & =X*W & \end{array}$$ (15)

In Eq (13), Eq (14) and Eq (15), *U*, *V* and *W* are convolution kernels with sizes 3×3, 5×5, and 7×7, respectively. These kernels are used to capture local details, medium-scale contextual information, and broader global context, respectively.

The final edge-weighted features are obtained by combining these multi-scale feature maps:


$$\begin{array}{ccc} EE-MSFF=\alpha Q_{Edge}+\beta K_{Edge}+\gamma Z_{Edge}+\delta M+\varepsilon N+\zeta P & & \end{array}$$ (16)

Where *α*,*β*,*γ*,*δ*,*ε* and *ζ* are weighting coefficients that adjust the contributions of the different features.

The output of the EE-MSFF module is a processed feature map with dimensions b×c×h×w, where each channel contains feature information extracted from different convolution kernels and layers.EE-MSFF with dimensions as follows:


$$\begin{array}{ccc} EE-MSFF\in {\mathbb{R}}^{b\;\times \;c\;\times \;h\;\times \;w} & & \end{array}$$ (17)

#### The structure of EE-MSFF block.

Residual networks (ResNet) have shown significant advantages in image recognition tasks [23]. However, networks of different depths extract features in different ways [24]. Shallow networks mainly focus on low-level features such as edges and textures, while deeper networks capture more high-level semantic features. In crack detection tasks, edge information and subtle texture variations are crucial for accurate crack identification. Based on this, ResNet18 was chosen as the base architecture to take full advantage of its strength in extracting low-level features, and integrated the EE-MSFF mechanism into the BasicBlock of ResNet18 to improve crack detection accuracy and performance.

**Fig 4:**
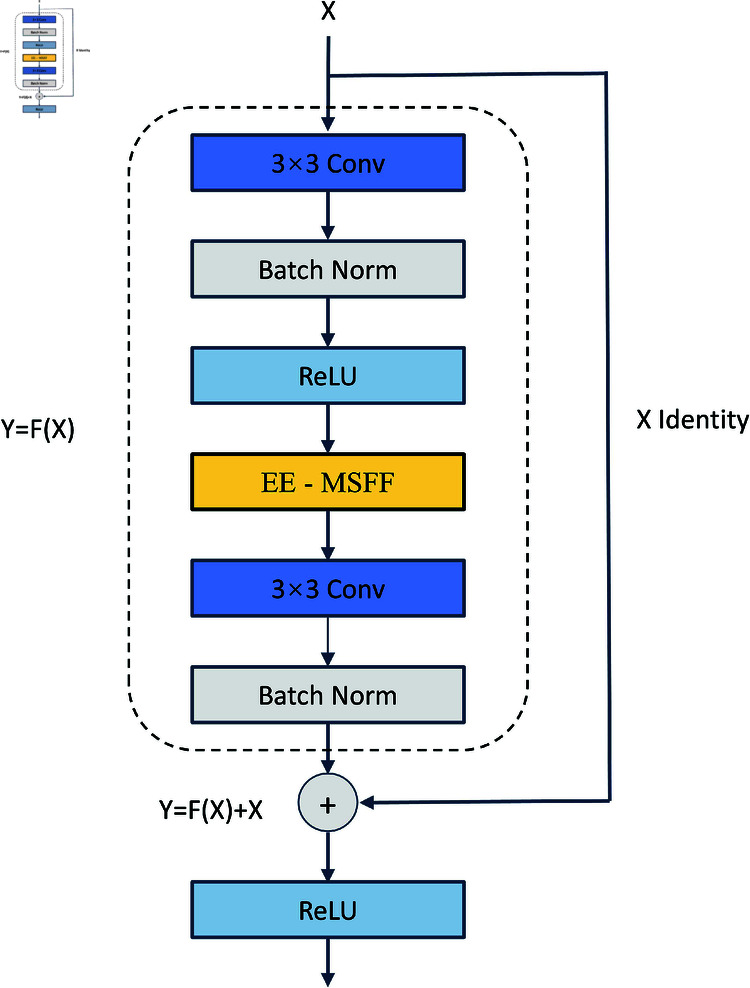
EE-MSFF block.

As shown in the Fig 4, the BasicBlock of ResNet18 consists of two convolutional layers and a residual connection. Before adding the EE-MSFF module, for a given input matrix *X*, the output matrix *F* is calculated using the following expression [25]:


$$\begin{array}{ccc} F=\omega_{2}*\sigma (\omega_{1}*X) & & \end{array}$$ (18)

Where *σ* represents the activation function *ReLU*, and *ω*_2_ denote the weights of the first and second convolutional layers, respectively.

Next, the matrix *F* is added to the input matrix *X* to get the final output matrix *Y*:


$$\begin{array}{ccc} Y=F(X)+X & & \end{array}$$ (19)

Through the residual block, the network learns incremental changes in the input information and adds it to the original input, effectively mitigating the vanishing gradient problem and improving the model’s training efficiency.

After embedding the EE-MSFF module between the two convolutional layers, the final output matrix *Y* can be represented by the following formula:


$$\begin{array}{ccc} Y=\omega_{2}*EE-MSFF*\sigma (\omega_{1}*X)+X & & \end{array}$$ (20)

## Results and discussion

The experiments were conducted using Python 3.7 and the PaddlePaddle 2.4.0 deep learning framework. The runtime environment was a Linux system equipped with a Tesla V100 GPU, 32GB of GPU memory, a 4-core CPU, and 32GB of RAM.

### Training parameters and model configuration

During the training process of the model, an initial batch size of 4 was set, with 200 epochs for training on the RDD2020 dataset and 40 epochs for training on the Concrete_Data_Week3 dataset. The model parameters were optimized using the Adam optimizer, and cross-entropy was used as the loss function to measure the discrepancy between the true values and the predicted results [26,27]. The learning rate was set at 0.01. Taking the RDD2020 dataset as an example, Table 3 provides detailed information on the parameter settings of each layer in the model structure.

**Table 3:** Model structure details.

Layers or Blocks	Input	EE-MSFF Information	Output
Input Layer	(4, 3, 224, 224)	-	(4, 3, 224, 224)
Convolutional Layer	(4, 3, 224, 224)	-	(4, 64, 224, 224)
EE-MSFF Block1	(4, 64, 224, 224)	(4, 64, 224, 224)	(4, 64, 224, 224)
ResNet Basic Block1	(4, 64, 224, 224)	-	(4, 64, 224, 224)
EE-MSFF Block2	(4, 64, 224, 224)	(4, 64, 224, 224)	(4, 64, 224, 224)
ResNet Basic Block2	(4, 64, 224, 224)	-	(4, 64, 224, 224)
ResNet Basic Block3	(4, 64, 224, 224)	-	(4, 128, 112, 112)
ResNet Basic Block 3	(4, 128, 112, 112)	-	(4, 256, 56, 56)
ResNet Basic Block 4	(4, 128, 112, 112)	-	(4, 256, 56, 56)
ResNet Basic Block 4	(4, 256, 56, 56)	-	(4, 256, 28, 28)
ResNet Basic Block 5	(4, 256, 56, 56)	-	(4, 512, 28, 28)
ResNet Basic Block 5	(4, 512, 28, 28)	-	(4, 512, 28, 28)
Avg Pooling Layer	(4, 512, 28, 28)	-	(4, 512,1, 1)
Flatten	(4, 512,1, 1)	-	(4, 512)
Fully Connected Layers	(4, 512)	-	(4,4)

### Model recognition results

**Fig 5:**
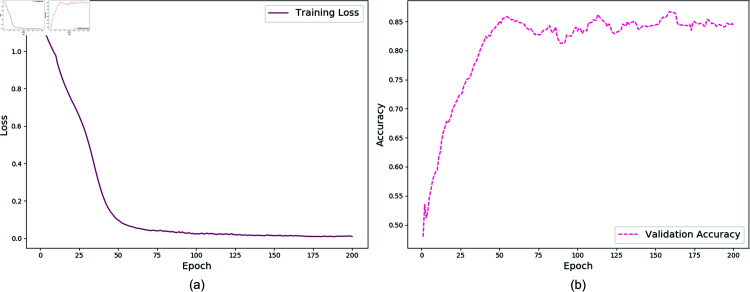
Training loss and validation accuracy on the RDD2020 dataset.

On the complex background dataset RDD2020, the model demonstrated effective recognition of various pavement defect types after 200 training epochs. The training loss converged to approximately 0.002, as shown in Fig 5(a), while the validation accuracy reached 88.68%, with the training progression illustrated in Fig 5(b).

**Fig 6:**
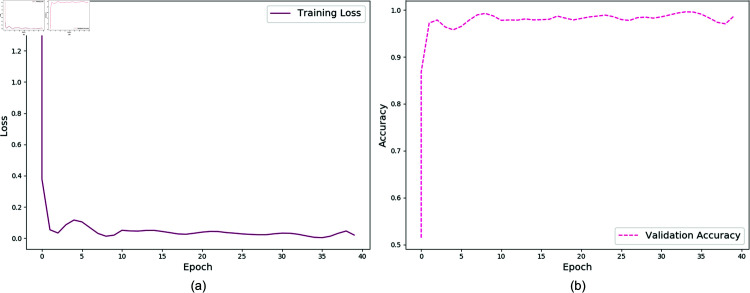
Training loss and validation accuracy on the Concrete_Data_Week3 dataset.

For the simple background dataset, Concrete_Data_Week3, the model was trained for 40 epochs, achieving a training loss of approximately 0.001, as depicted in Fig 6(a). The validation accuracy also reached 99.50%, with the corresponding training progression displayed in Fig 6(b). This result suggests that the proposed model is reasonably well-suited for datasets with simpler backgrounds and fewer defect categories, demonstrating its capability to adapt and learn effectively under such conditions.

**Fig 7:**
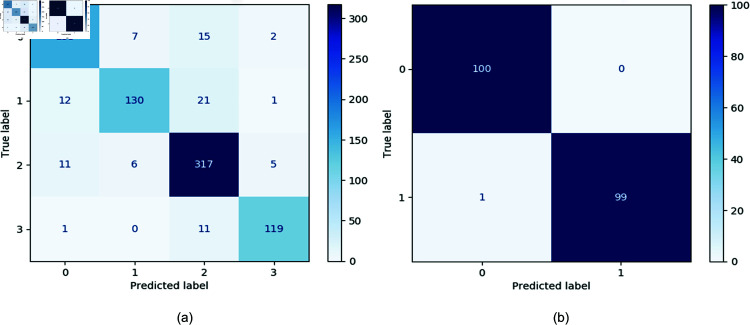
Confusion matrix for the result. Group a is the RDD2020 dataset, and Group b is the Concrete_Data_Week3 dataset.

The classification results on the RDD2020 dataset are detailed in the confusion matrix presented in Fig 7(a). A thorough analysis of this confusion matrix revealed that the model exhibited considerable misclassification in the second category, Transverse Cracks (D10), which corresponds to the class with the fewest samples.

For the Concrete_Data_Week3 dataset, the confusion matrix shown in Fig 7(b) demonstrates that our model performed relatively well in classifying a small-scale dataset with simple backgrounds. These findings highlight the model’s promising learning capabilities in environments with lower complexity.

### Ablation study

Ablation experiments were conducted on the modules used in the study to validate the effectiveness of the proposed approach. The experimental subjects included the baseline ResNet18 module and the EE - MSFF module designed in this study. The results are shown in Table 4.

In this study, the performance metrics used include Accuracy, Precision, Recall, and F1-Score, with their mathematical definitions as follows:

- Accuracy: Measures the overall correctness of the model’s predictions. The formula is given by:
  $$\begin{array}{ccc} Accuracy=\frac{TP+TN}{TP+TN+FP+FN} & & \end{array}$$ (21)
  where TP represents True Positives, TN represents True Negatives, FP represents False Positives, and FN represents False Negatives.
- Precision: Focuses on the proportion of true positives among all samples predicted as positive.The formula can be expressed as:
  $$\begin{array}{ccc} Precision=\frac{TP}{TP+FP} & & \end{array}$$ (22)
- Recall: Indicates the proportion of actual positives correctly identified by the model. Its formula is as follows:
  $$\begin{array}{ccc} Recall=\frac{TP}{TP+FN} & & \end{array}$$ (23)
- F1-Score: Combines Precision and Recall into a single metric, emphasizing their harmonic mean. The formula for calculating the F1-Score is:

**Table 4:** Ablation study data comparison.

Model	Accuracy (%)	Precision (%)	Recall (%)	F1-Score (%)
ResNet18	86.34	86.55	85.80	86.12
ResNet18+EE-MSFF	88.68	89.57	87.55	88.42


$$\begin{array}{ccc} F1\text{-}Score=\frac{2\times Precision\times Recall}{Precision+Recall} & & \end{array}$$ (24)

These metrics collectively provide a comprehensive evaluation of the model’s performance.

Through a series of ablation experiments, it was observed that incorporating the EE-MSFF mechanism positively influenced model accuracy, leading to an improvement of 2.34%. Other metrics, including Precision, Recall, and F1-Score, also exhibited enhancements, indicating that the proposed model significantly improved both feature learning capabilities and overall generalization performance.

**Fig 8:**
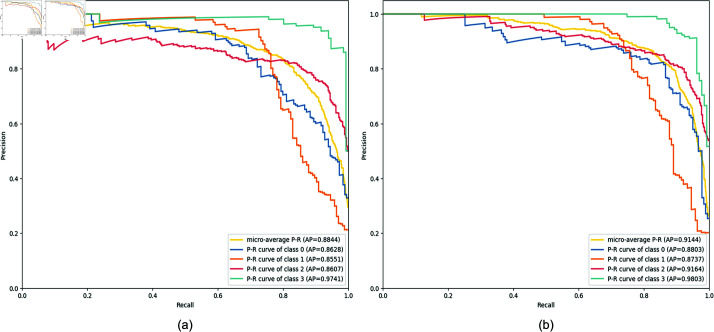
The precision-recall curve. Group a is the ResNet18 model, and Group b is the ResNet18 + EE-MSFF model.

To comprehensively evaluate the performance of the model, the Precision-Recall (P-R) curve was plotted, as shown in Fig 8. These curves provide a clearer view of the convergence trends for various metrics. The model not only maintains a high level of accuracy but also demonstrates favorable convergence in terms of precision, recall, and F1-Score.

**Fig 9:**
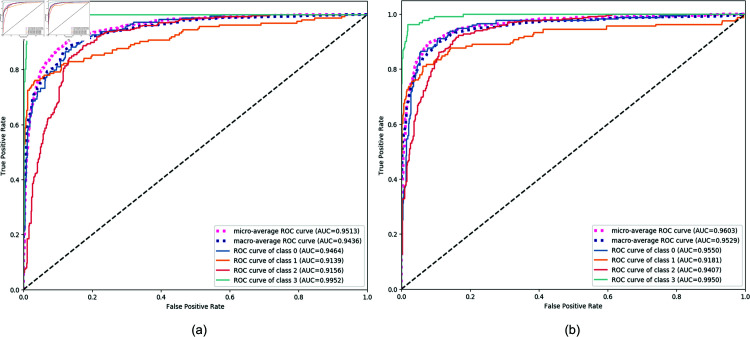
The ROC curve. Group a is the ResNet18 model, and Group b is the ResNet18 + EE-MSFF model.

The Receiver Operating Characteristic (ROC) curve was also plotted [28], as shown in Fig 9. The results indicate that the model converges slightly faster, with smoother transitions and minimal fluctuations.

However, based on the trends of the P-R and ROC curves, as well as feedback from related metrics, the classification performance for the second category is suboptimal. This can be attributed to the limited number of images in the second category, which constrains feature representation and subsequently impacts classification accuracy during training.

### Analysis of weighting parameters

**Fig 10:**
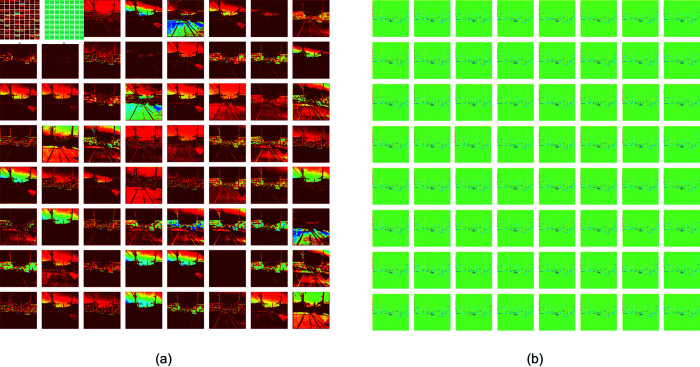
Comparison of 64-channel output heatmaps during model training. Group a is the heatmap before EE-MSFF optimization. Group b is the heatmap after EE-MSFF optimization. Republished from Reckless_Raccoon, RDD_2020 Dataset, which is available under a CC0 1.0 Universal Public Domain Dedication. The dataset can be accessed at: https://www.kaggle.com/datasets/recklessraccoon/rdd2020-yolov8-annotated-image-after/data.

To systematically demonstrate the impact of the EE-MSFF module on image processing, this study employed heatmap visualization techniques to analyze the distribution of model weights. [29] Specifically, during the training process, we compared the 64-channel output heatmaps at two stages: one without the EE-MSFF module, as shown in Fig 10(a), and one after optimization with the EE-MSFF module, as shown in Fig 10(b). The comparison clearly reveals that the model weights after EE-MSFF optimization are significantly concentrated around the edge regions of the image.

**Fig 11:**
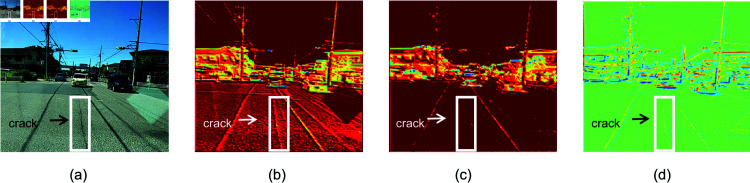
Detail comparison. Group a is the original images. Groups b and c is the heatmap before EE-MSFF optimization. Group d is the heatmap after EE-MSFF optimization. Republished from Reckless_Raccoon, RDD_2020 Dataset, which is available under a CC0 1.0 Universal Public Domain Dedication. The dataset can be accessed at: https://www.kaggle.com/datasets/recklessraccoon/rdd2020-yolov8-annotated-image-after/data.

For instance, when processing a road surface distress image, as shown in Fig 11(a), the model’s training weights, displayed in Figs 11(b) and Fig11(c), demonstrate insufficient focus on the road crack. In contrast, the model optimized with the EE-MSFF module, shown in Fig 11(d), clearly focuses more on the edge areas of the image, effectively preventing the omission of distress information and improving the model’s accuracy and robustness in road damage detection.

**Fig 12:**
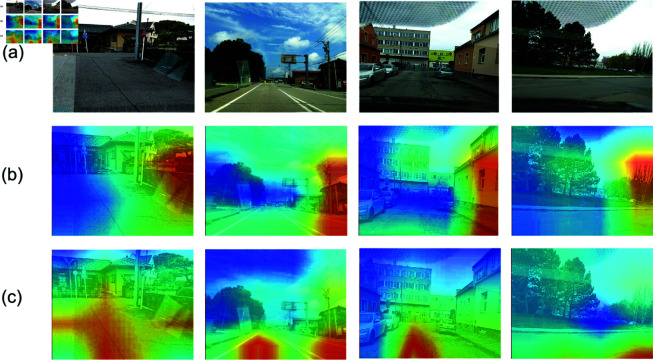
Heatmaps comparison. Group a is the original images. Group b is the heatmap of the ResNet18 model. Group c is the heatmap of the ResNet18 + EE-MSFF model. Republished from Reckless_Raccoon, RDD_2020 Dataset, which is available under a CC0 1.0 Universal Public Domain Dedication. The dataset can be accessed at: https://www.kaggle.com/datasets/recklessraccoon/rdd2020-yolov8-annotated-image-after/data.

To assess the final trained model’s performance, we performed predictive analysis on four typical road surface distress types, as shown in Fig 12(a). The results indicate that the model without the EE-MSFF module exhibited an attention mechanism biased toward background features, as shown in Fig 12(b), which could lead to the neglect of critical distress information.

In contrast, the model incorporating the EE-MSFF module demonstrated enhanced prediction accuracy, effectively focusing on different types of cracks, as shown in Fig 12(c). This improvement not only strengthened the model’s attention to target distress areas but also enhanced its ability to distinguish important features from irrelevant background noise. Therefore, it is evident that the EE-MSFF module optimized the model’s attention distribution, ensuring high sensitivity and specificity in road distress detection, thereby providing more reliable technical support for subsequent road maintenance decisions.

### Comparison

With the development of convolutional neural networks, models such as VGG16, GoogLeNet, and MobileNet_V2 have demonstrated solid performance in various image classification tasks, thanks to their efficient feature extraction capabilities, making them well-established choices in the field. Experiments were conducted on the RDD2020 dataset with complex backgrounds to assess the performance of the proposed model in these tasks. The comparison results are summarized in Table 5.

From Table 5, the proposed model demonstrates improved accuracy over other classical models, with gains of approximately 1.10% to 2.30%, except for ResNet34. Although the accuracy of the model is 0.37% lower than ResNet34, the performance gap is narrowed by achieving a 2.34% increase in accuracy compared to ResNet18. This result suggests that the integration of edge-enhanced features contributes to performance improvements, even with a shallower network structure.

In terms of precision, the model surpasses other classical models by approximately 2.00% to 3.00%, with a modest increase of 0.06% over ResNet34. This outcome indicates that the proposed approach enhances detection reliability without necessitating deeper network architectures. Precision improvements are particularly valuable in practical pavement damage detection, as they help minimize false positives, leading to more reliable identification of actual defects.

**Table 5:** Classification data comparison of classical models on the RDD2020 dataset.

No.	Model	Accuracy (%)	Precision (%)	Recall (%)	F1-Score (%)
1	ResNet34	89.05	89.51	88.35	88.80
2	VGG16	87.57	87.39	87.49	87.43
3	GoogLeNet	87.30	87.12	87.21	87.16
4	MobileNet_V2	87.20	87.67	86.09	86.76
5	ResNet18	86.34	86.55	85.80	86.12
**6**	**ResNet18+EE-MSFF**	**88.68**	**89.57**	**87.55**	**88.42**

The incorporation of edge-enhanced features contributes to performance improvements by enabling the network to focus on fine-grained details essential for damage identification. Although ResNet34 achieves slightly higher accuracy, the proposed model delivers comparable results with a shallower architecture, demonstrating the effectiveness of feature enhancement without relying solely on increased network depth. This balance between accuracy and precision underscores the practical benefits of the approach, particularly in applications where reliable defect detection is critical.

Furthermore, the proposed model was compared with the state-of-the-art DeiT model [16] on the simple-background dataset Concrete_Data_Week3, which contains fewer images. Previous studies showed that the DeiT model performed relatively well compared to other models. To ensure a fair comparison, experiments were conducted in the same environment. As shown in Table 6, the proposed model achieved an accuracy of 99.50%, with only one misclassified image—similar to the results of the DeiT model.

**Table 6:** Comparison of classification data for various models on the Concrete_Data_Week3 dataset [16].

No.	Model	Accuracy (%)	Precision (%)	Recall (%)	F1-Score (%)
1	Xception	93.21	94.61	91.80	93.10
2	ResNet50	88.00	89.58	86.00	87.75
3	YOLOv5	95.00	93.69	96.50	95.07
4	YOLOv8	96.50	97.44	95.50	96.46
5	MobileNet_V2	98.00	99.48	96.50	97.96
6	DeIT	99.50	99.50	100	99.50
7	ResNet18	98.00	98.07	98.00	97.99
**8**	**ResNet18+EE-MSFF**	**99.50**	**100**	**99.50**	**99.50**

In addition to achieving high accuracy, the proposed model attained 100% precision under simple background conditions, indicating its effectiveness in reducing false positives. These results suggest that while the model performs well in complex environments, it also delivers strong performance in simpler scenarios, reflecting its adaptability and potential for broader application across different datasets.

## Conclusion

This study explores the enhancement effect of edge detection operators in pavement crack detection tasks by introducing a multi-scale feature fusion mechanism. This approach improves the model’s ability to perceive and represent crack features, leading to favorable crack recognition performance under both complex and simple backgrounds. The proposed EE-MSFF mechanism leverages edge information during the feature extraction stage by applying Sobel, Prewitt, and Laplacian operators to extract and fuse multi-scale edge features from images. This enhances the model’s sensitivity to fine-grained crack features and contributes to greater robustness and generalization in the presence of complex backgrounds.

In the experimental phase, systematic ablation studies were conducted to quantify the independent contribution of the EE-MSFF module to overall performance. Additionally, the proposed model was compared with classical convolutional neural network models, including ResNet18, VGG16, and GoogLeNet, as well as advanced models such as YOLOv5 and YOLOv8, to evaluate its effectiveness and competitiveness. The experimental results show that the model achieved an accuracy of 88.68% on the RDD2020 dataset with complex backgrounds and 99.5% on the Concrete_Data_Week3 dataset with simpler backgrounds. The ablation studies indicate that the EE-MSFF module enhances the model’s ability to represent crack features, contributing to a 2.34% improvement in classification accuracy compared to the baseline ResNet18 model, while helping to reduce false positives and false negatives in complex environments.

This study integrates traditional image processing methods with deep convolutional neural network techniques, utilizing multi-scale edge features to explicitly guide the detection of crack regions. By increasing the model’s attention to crack areas during training, the approach enhances accuracy and robustness in pavement distress detection tasks. This method shows potential value and practical significance for road inspection and maintenance, offering municipal authorities and transportation agencies an efficient and cost-effective solution for automated pavement distress detection.

In future research endeavors, the intention is to enhance the model architecture by integrating advanced methodologies like attention mechanisms. This modification is designed to augment the model’s proficiency in capturing intricate crack details within complex surroundings. Additionally, efforts will focus on developing lightweight models to accommodate embedded devices and edge computing applications, supporting broader deployment of pavement distress detection technology in real-world engineering scenarios.
